# Hyperbranched Macromolecules: From Synthesis to Applications

**DOI:** 10.3390/molecules23030657

**Published:** 2018-03-14

**Authors:** In-Yup Jeon, Hyuk-Jun Noh, Jong-Beom Baek

**Affiliations:** 1 Department of Chemical Engineering, Wonkwang University, 460, Iksandae-ro, Iksan, Jeonbuk 54538, Korea; 2 School of Energy and Chemical Engineering/Center for Dimension-Controllable Organic Frameworks, Ulsan National Institute of Science and Technology (UNIST), 50, UNIST, Ulsan 44919, Korea; hyukjun93@unist.ac.kr

**Keywords:** hyperbranched macromolecules, polymerization, photoelectric materials, stabilizers, bio-applications, carbon nanomaterial

## Abstract

Hyperbranched macromolecules (HMs, also called hyperbranched polymers) are highly branched three-dimensional (3D) structures in which all bonds converge to a focal point or core, and which have a multiplicity of reactive chain-ends. This review summarizes major types of synthetic strategies exploited to produce HMs, including the step-growth polycondensation, the self-condensing vinyl polymerization and ring opening polymerization. Compared to linear analogues, the globular and dendritic architectures of HMs endow new characteristics, such as abundant functional groups, intramolecular cavities, low viscosity, and high solubility. After discussing the general concepts, synthesis, and properties, various applications of HMs are also covered. HMs continue being materials for topical interest, and thus this review offers both concise summary for those new to the topic and for those with more experience in the field of HMs.

## 1. Introduction

Dendritic macromolecules have unique architectures quite unlike their linear, branched, and crosslinked analogues. Dendritic macromolecules are classified as dendrons, dendrimers, or hyperbranched macromolecules (HMs, also called hyperbranched polymers), all of which are composed of successive branching units. Dendritic macromolecules have attracted considerable attention during recent decades, because of their unusual properties, such as low viscosity, high solubility, and high functionality (Table 1). These properties stem from their globular and spherical molecular architectures. 

A dendrimer consists of two types of structural units: uniform terminal units on the globular surface and dendritic units inside. Thus, dendrimers have well-defined molecular weights with unique symmetric structures. The main drawback for practical applications of dendrimers is the tedious stepwise synthesis required, along with time-consuming purification at each step. Consequently, more efficient methods for production of dendritic macromolecules should involve less tedious synthesis procedures. This is possible by forming hyperbranched macromolecules (HMs). While dendrimers have well defined structure and molecular weight, HMs consist of a mixture of linear and branched units inside with multifunctional groups on their periphery. They still possess a highly branched architecture with a three-dimensional globular shape. The structural difference between dendrimers and HMs is ascribed to the difference in their formation mechanism; thus, it can be further related to their different synthetic approaches used for them. In the case of HMs, their termini are located on the periphery, which is similar to dendrimers. However, the structure of the former is irregular, because linear and branched units are randomly distributed within the macromolecular framework (or polymer backbone). In brief, HMs have more irregular structures with polydispersity of molecular weight than do dendrimers, which have perfect structures with monodispersity of molecular weight [1,2,3,4,5,6]. Nevertheless, HMs have demonstrated several characteristics similar to those of dendrimers, including multifunctionality on their periphery, low solution (melt) viscosity, and better solubility [4]. This section focuses on the synthesis, properties, and the applications of HMs developed during the last decades.

## 2. Synthesis of HMs

There are three main approaches to the synthesis of HMs: (i) step-growth polycondensation of AB_x_ (x ≥ 2) or A_2_ + B_3_ monomers, (ii) self-condensing vinyl polymerization, and (iii) ring-opening polymerization [8]. 

### 2.1. Step-Growth Polycondensation 

This strategy involves the polymerization of AB_x_ (x ≥ 2) monomers via one-step polycondensation [9,10,11,12,13,14,15]. The primary advantage of this approach is that normal step-growth polymerization characteristics are obeyed. However, the main drawbacks include gelation, which often occurs during the polymerization. A monomer with functionality of three or more can form HMs and can fast reach gel point forming a cross-linked network structure even at low fractional conversion. The conversion, at which a tree-like topology turns into a network structure, is known as a gel point. The step-polymerization can be simply quenched the reaction prior to reach the gel point. Still, the purification is required to exclude minor cross-linked structures, and thus to afford pure desired HMs. 

Another drawback is that the AB_x_ monomers employed have to be synthesized prior to polymerization and this is a distinct disadvantage for commercial applications. However, the step-growth polycondensation process offers diverse synthesis of HMs using a variety of available monomers, which provides the potential for preparation of a wide spectrum of functionalities. 

**Scheme 1:**
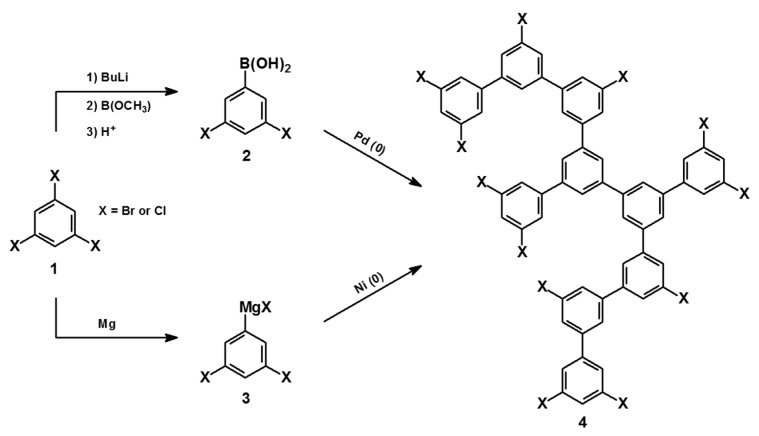
Synthesis of HMs via step-growth polycondensation. Reproduced from [9,10,11] with permission from the American Chemical Society.

AB_2_-type monomers are often used as building blocks, due probably to their easy synthesis (Scheme 1), while the other AB_x_ (x ≥ 3) monomers have been reported for use in the preparation of hyperbranched polyesters [15,16] and polysiloxanes [17]. For example, 5-acetoxyisophthalic acid was used as the AB_2_ monomer in melt polymerization to prepare hyperbranched aromatic polyesters that were insoluble in organic solvents. This was due to intermolecular dehydration, which occurred between the carboxylic acid groups during melt polymerization. However, hydrolysis of the crude product produced a soluble hyperbranched polyester with a large number of carboxylic acid groups [18]. Aromatic-aliphatic hyperbranched polyethers were also prepared by forming benzyl ether linkages in the presence of K_2_CO_3_ and crown ether (18-crown-6) in acetone [19].

### 2.2. Self-Condensing Vinyl Polymerization

**Scheme 2:**
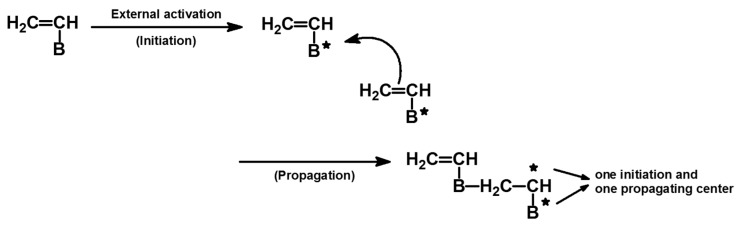
Synthesis of HMs via self-condensing vinyl polymerization. Reproduced from [21] with permission from the Springer Nature.

Self-condensing vinyl polymerization was defined by Fréchet et al. [20]. This process involves the use of monomers that feature one vinyl group and one initiating moiety (AB* monomers) to generate HMs (Scheme 2). The activated species can be a radical, cation, or even a carbanion. 

After the initiating moiety is activated, it is reacted with a vinyl group to form a covalent bond and a new active site on the α-carbon atom of the double bond. The number of activation sites increases in proportion with the propagation reaction in self-condensing vinyl polymerization, whereas two functional groups are always consumed during polymerization. Therefore, in this process, living/controlled polymerization systems are preferred in order to avoid crosslinking reactions (i.e., gelation) caused by dimerization or chain-transfer reactions.

### 2.3. Ring-Opening Polymerization

**Scheme 3:**
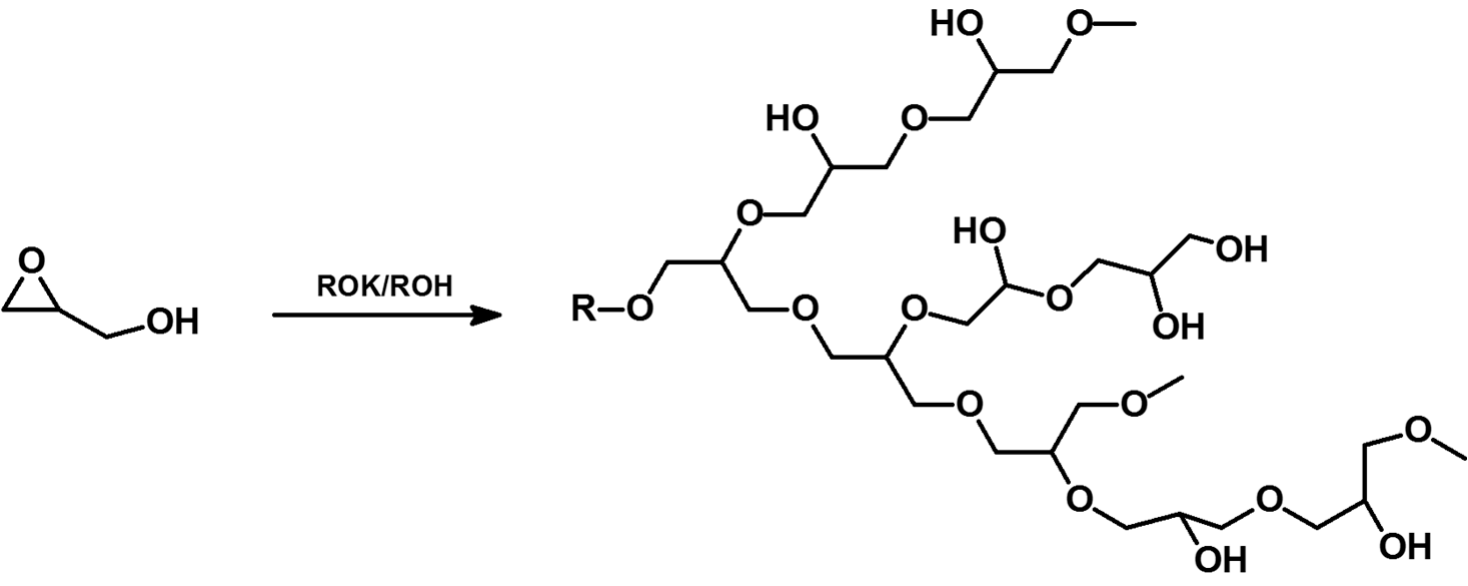
Synthesis of HMs via ring-opening polymerization. Reproduced from [21] with permission from the Springer Nature.

The third approach is called ring-opening polymerization (Scheme 3). Although the monomer itself does not contain branching points, these are generated through the propagation reaction, similar to that in the self-condensing vinyl polymerization (Scheme 2). Therefore, the monomer can be considered a latent AB_x_ monomer. Polymerization is driven by addition of a proper initiator to the corresponding monomer. As an example, anionic ring-opening polymerization of glycidol was used to prepare hyperbranched aliphatic polyether that contained one epoxide and one hydroxy group, representing a latent AB_2_ monomer [22].

### 2.4. Alternative Routes for HMs

In addition to the three main routes discussed to prepare HMs, there are a few notable variants that merit discussion. As a consequence of the infrequent commercial availability of AB_2_ monomers, other researchers have begun to focus on polycondensation of A_2_ and B_3_ monomers (the A_2_ + B_3_ route). Generally, the success of this approach is dependent upon many factors, including the ratio of functionalities, solvent and reagent purity, and the reaction time and temperature (conversion). This type of approach is obviously difficult to control and the resultant HMs often have high molecular masses upon gelation [23,24,25].

Other approaches led to polymers with topologies similar to that of comb or star shaped polymer architectures. The issue of polymerization control has proven to be paramount. In the case of the ‘graft onto’ approach, although steric and dilution effects limit the size of the polymers, they possess a high degree of branching. In the case of the ‘graft from’ approach, a high degree of control over the polymer architecture were obtained. A ‘graft onto’ polymerization was reported in 1991 [26]. Using the polyoxazoline approach, comb-burst poly(ethylenimine)-poly 2-ethyl-2-oxazoline copolymers, and poly(ethylenimine) homopolymers were produced. In contrast, the ‘graft from’ approach described, was used to form branched copolymers utilizing ‘living’ free radical polymerization in 1997 [27]. This approach was utilized to afford a wide variety of complex architectures in relatively few steps from commercially available monomers.

## 3. Properties of HMs

The physical properties of HMs are of key importance for their implementation in industrial applications. The viscosity of HMs, in both solution and molten states, has been found to be considerably lower than for their linear analogues [8,28]. Low-viscosity is one of the most interesting features of HMs, along with very good solubility in various solvents. 

### 3.1. Solubility

**Figure 1:**
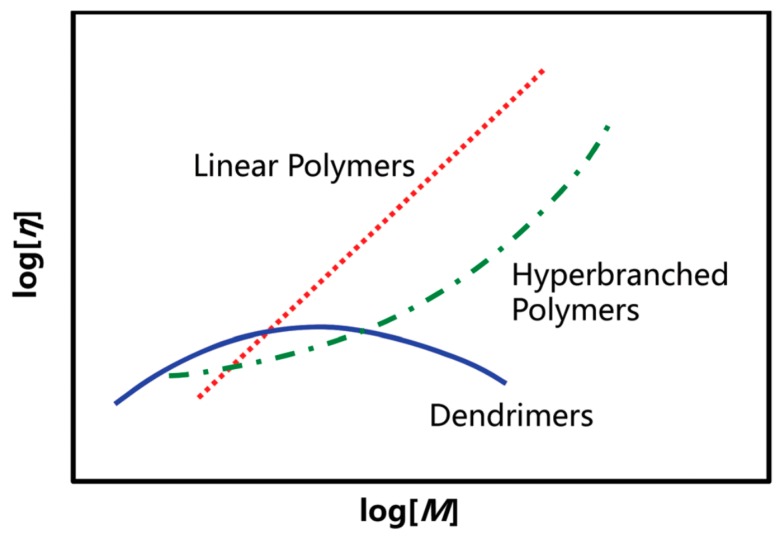
Relationship between log MW and log [η] for linear, dendrimer, and hyperbranched molecules. Reproduced from [31] with permission from the Royal Society of Chemistry.

The high solubility of HMs induced by a branched backbone is one important way that they differ from the linear polymers. Kim and Webster reported that hyperbranched polyphenylenes [11] had much better solubility in various solvents than did linear polyphenylenes. The solubility and solution behavior of HMs differ from those of linear ones. It is well known that the solution viscosity of dendritic macromolecules is lower than that of conventional linear polymers [18,29,30]. Such low viscosity indicates that dendritic macromolecules are less entangled due to their unique spherical shape. The relationship between intrinsic viscosity and molecular weight (MW) is shown in Figure 1. Dendrimers display a bell-shaped relationship, resulting from their well-defined globular structures. On the other hand, the intrinsic viscosity of HMs increases with MW, and the slopes of their plots are much lower than those of linear polymers. Moreover, the size exclusion chromatography (SEC) measurements indicated that the retention volume for HMs tended to be greater than that of linear polymers, when compared with the same MWs. The results suggested more compact conformation of HMs than of linear polymers in a solution. 

### 3.2. Thermal Properties

HMs are mostly amorphous materials, though some exceptional examples have been reported. For example, HMs have been modified to induce liquid crystallinity [32,33] or crystallinity [34]. The lower glass transition temperature (T_g_) of HMs than of linear polymers is another important feature. The glass transition behavior is related to the relatively large segmental motions within the polymeric frameworks, and the role of the end groups can be disregarded above a certain MW of a linear polymer. However, in the case of HMs, the segmental motions are strongly affected by the branching points, which induce large free volume, as well as the presence of abundant end groups. Therefore, the glass transition for HMs is strongly affected by the translational movement of the entire molecule instead of segmental movements [11,35]. Moreover, the chemical nature of HMs has a decisive effect on T_g_. For example, an aliphatic polyester generally has a much lower T_g_ value than an aromatic polyester having the same MW [35].

### 3.3. Mechanical Properties

Mechanical properties (e.g., initial modulus, tensile strength, compressive modulus) reflect the highly branched, compact structures of these relatively new polymer architectures [36,37]. The less or non-entangled state of HMs imposes rather poor mechanical integrity, sometimes resulting in brittleness. These features of HMs have limited their use in thermoplastics, in which mechanical strength is of importance. However, HMs can be used as additives for modification of viscosity to enhance the processability of thermoplastics. 

## 4. Structure of HMs

### 4.1. Degree of Branching (DB)

**Figure 2:**
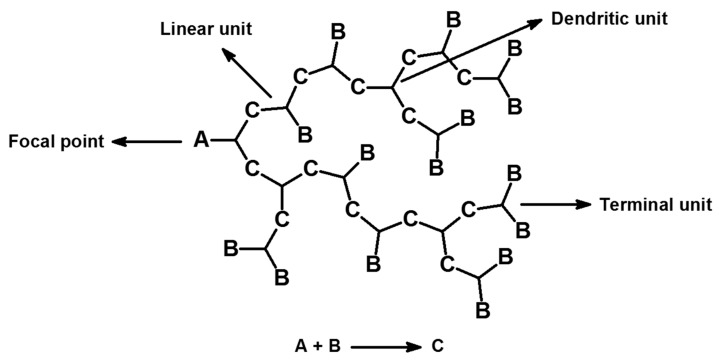
Different segment types in HMs.

A perfectly branched dendrimer is composed of two types of structural units: terminal units on the globular surface and dendritic units inside. On the other hand, HMs possess three types of structural units as illustrated in Figure 2: dendritic unit (D = fully incorporated with AB_x_ monomer), terminal units (T = two unreacted B groups), and linear units (L = one unreacted B group). The linear segments are generally described as defects. Fréchet et al. [38] defined the term ‘degree of branching’ (DB) as:

DB = (D + T)/(D + L + T)(1)

where D, T, and L are the number of dendritic, terminal, and linear units, respectively. DB is one of the important characteristics that indicate the branching structure of HMs. Frey and colleagues [39] reported a modified definition of DB based on the growth directions as:

DB = 2D/(2D + L) = (D + T − N)/(D + L + T − N)(2)

where N is the number of molecules. The two Equations give almost the same DBs for HMs with high MWs. This is because the N in Frey’s equation is negligible in such cases.

The DB of HMs can be measured via direct and indirect methods. The direct methods include NMR measurements and degradation of the polymer units. The model compounds need to be characterized by ^13^C-nuclear magnetic resonance (NMR). On the basis of ^13^C-NMR spectra, different peaks from the different branching units of HMs can be assigned. DB can also be calculated from integrals of the corresponding peaks [38]. In addition, an indirect method based on degradation of the hyperbranched backbone was introduced by Kambouris and Hawker [40]. The chain ends are chemically modified and then the hyperbranched skeleton is fully degraded by hydrolysis. The degradation products are identified using capillary chromatography. To use this technique successfully, there are two prerequisites. First, the chain ends must remain intact during the degradation, and second, conversion to elementary subunits must be complete [40].

DB can be altered or tuned to some extent [41,42,43,44,45,46] via four major methods: (i) copolymerization of AB_2_ and AB monomers with different feed ratios [13]; (ii) changing the polymerization conditions such as temperature, the ratio of monomer to catalyst and solvent [47,48,49,50], and the monomer pressure [51,52]; (iii) host-guest inclusion of AB_2_ or a multifunctional monomer [53]; and (iv) combinations of these three. Moreover, five methods have been tried to increase DB: (i) increasing the reactivity of the B′ group (residual functional group on the linear unit) [54], (ii) addition of core molecules [55], (iii) polycondensation of dendrons [56], (iv) post-modification of the formed HMs to convert the linear units to dendritic ones [57], and (v) using a special catalyst [58]. 

### 4.2. Molecular Weight

Molecular weight (MW) and the polydispersity index (PDI) are significant parameters for determining the characteristics of HMs. Based on statistical and kinetic methods for HMs prepared by the polycondensation of AB_x_ (x ≥ 2) monomers, DP and PDI depend on conversion of the monomers [59,60]. Obviously, PDI increases with increasing conversion. Nevertheless, in some experiments, PDI could be narrowed by utilizing specific techniques, including: (i) slow addition of monomers [61,62,63,64,65], (ii) copolymerization with core molecules [55,63,64,65,66,67], and (iii) separation by dialysis or precipitation [68].

## 5. Potential Applications of HMs

**Figure 3:**
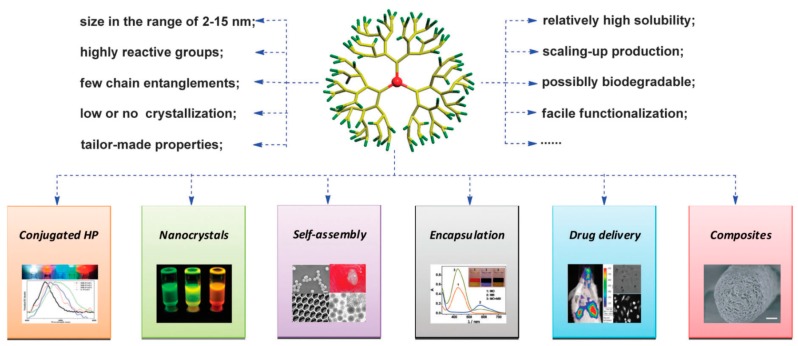
Relationship between the structure and properties of HMs and their major applications. Reproduced from [7] with permission from the Royal Society of Chemistry.

Generally, in comparison with linear analogues, HMs display many peculiar features, such as large number of reactive end-groups, few chain entanglements, and little or no crystallization (amorphous). The new properties allow them to provide new features such as large free volume, tailor-made properties, enhanced solubility, and low viscosity. To tune their properties, it gives rise to diverse HMs with desirable functional groups (e.g., –COOH, –OH, –NH_2_, O=C–NH_2_, etc.) and topologies such as segmented or sequential units. Benefiting from tunable nature and correspondingly new properties, the produced HMs have been widely applied in various new fields, including photoelectronics, nanotechnology, biomedicine, composites, coatings, adhesives, and modifiers (Figure 3). 

### 5.1. Photoelectric Materials

When compared with linear polymers, conjugated HMs (CHMs) have better solubility and processability. Moreover, their highly branched and globular frameworks can prevent aggregation and reduce interunit reactions. Driven by the requirement for unusual properties, much effort has been devoted to the design and synthesis of CHMs. 

With donor-π-acceptor chromophores, non-linear optical (NLO) materials play a significant role in latent electro-optic applications [69]. For high performance NLO materials, one of the daunting problems is how to eliminate intermolecular dipole-dipole interactions. Such defects can be efficiently restrained by building chromophores in the main-chain [70,71], side-chain [72,73], and periphery [74] of HMs. 

To prevent undesired dipole-dipole interactions, direct polycondensation through an A_2_ + B_4_ route using Suzuki coupling reaction has been applied for the synthesis of soluble HMs (two hyperbranched NLO polymers HP1 and HP2) with isolated chromophores [70]. HP1 and HP2 from A_4_ + B_2_ (boronic ester) monomers, containing nitro-based chromophore and sulfonyl-based chromophore, were also prepared via click reaction. According to second harmonic generation measurements, the d_33_ coefficients were 40.0 and 73.6 pm V^−1^ with *Φ* values of 0.11 and 0.13. Peripheral chromophore-modified HMs can also reduce the dipole-dipole interactions. Although the content of such a chromophore is lower (~20–23 wt %) than that of their linear polymers, the d_33_ coefficients are similar (up to 65 pm V^−1^). The result can be attributed to their unique molecular architectures [75].

**Figure 4:**
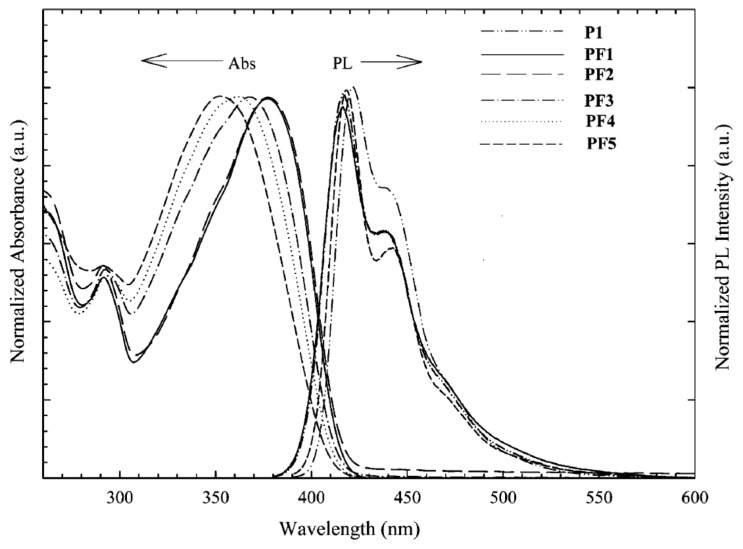
Photoluminescence and absorption spectra of HPFs in CHCl_3_. Reproduced from [78] with permission from the American Chemical Society.

Among the diverse CHMs, polyfluorines (PFs) are very important candidates for blue light emitting diodes (LEDs) due to their desirable luminous intensity [76,77,78,79,80,81,82,83]. To reduce detrimental green emission and/or inherent ketonic defects, the incorporation of triazole, truxene, oxadiazole, or carbazole building units into hyperbranched polyfluorines (HPFs) has been used to improve their electron transport capabilities. A series of novel HPFs were prepared using Suzuki cross-coupling [78]. The resultant products were soluble in common organic solvents (i.e., CHCl_3_, CH_2_Cl_2_, and toluene) and displayed good thermal stability. Either in film or in chloroform solution, they exhibited absorption maxima at 349–378 nm (Figure 4). For an LED using HPF as the emitting layer, the blue emission was up to 212 cd m^−2^ at about 19 V.

### 5.2. Stabilizers for Nanocrystals

Nanocrystals (NCs or nanoparticles) include insulator, semiconductor, and metal crystals that show unique size-dependent physical or chemical properties [84,85]. Spontaneous aggregation of NC particles leads to degradation of performance. Therefore, to minimize the problem, HMs are often used as stabilizers in the preparation of NCs due to their special characteristics, such as their specific three-dimensional structure, good solubility, and lots of intramolecular hollow space (free-volume). The influence of the HM structure on the synthesis of NCs is mainly shown in the following three aspects: (i) their unique 3D structure can provide sufficient hindrance, and thus can efficiently suppress the aggregation tendency of NCs, (ii) the presence of many cavities in the HM templates confines the free diffusion of NC precursors, and hence are useful for controlling the size of NC particles, and (iii) the terminal groups of HMs provide enough functional flexibility to facilitate the synthesis and dimensional control of NC particles. 

**Figure 5:**
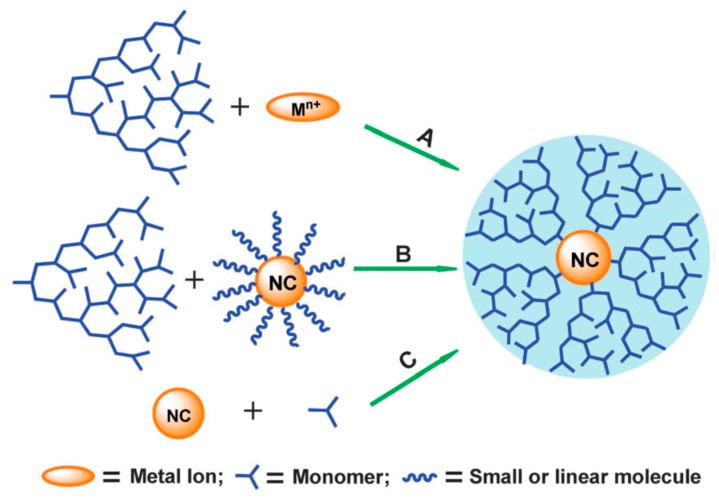
HMs as stabilizer for nanocrystals (NCs): (A) HMs first, (B) ligand exchange, and (C) NCs first. Reproduced from [7] with permission from the Royal Society of Chemistry.

Three methods have been reported for the synthesis of NCs: (i) HMs first (HMs use as stabilizers to directly prepare NCs); (ii) ligand exchange (NCs-coated surfactants or linear polymers as ligands are exchanged into an appropriate HMs); and (iii) NCs first (the grafting or in-situ growth of HMs occurs on the surface of NCs) (Figure 5).

**Figure 6:**
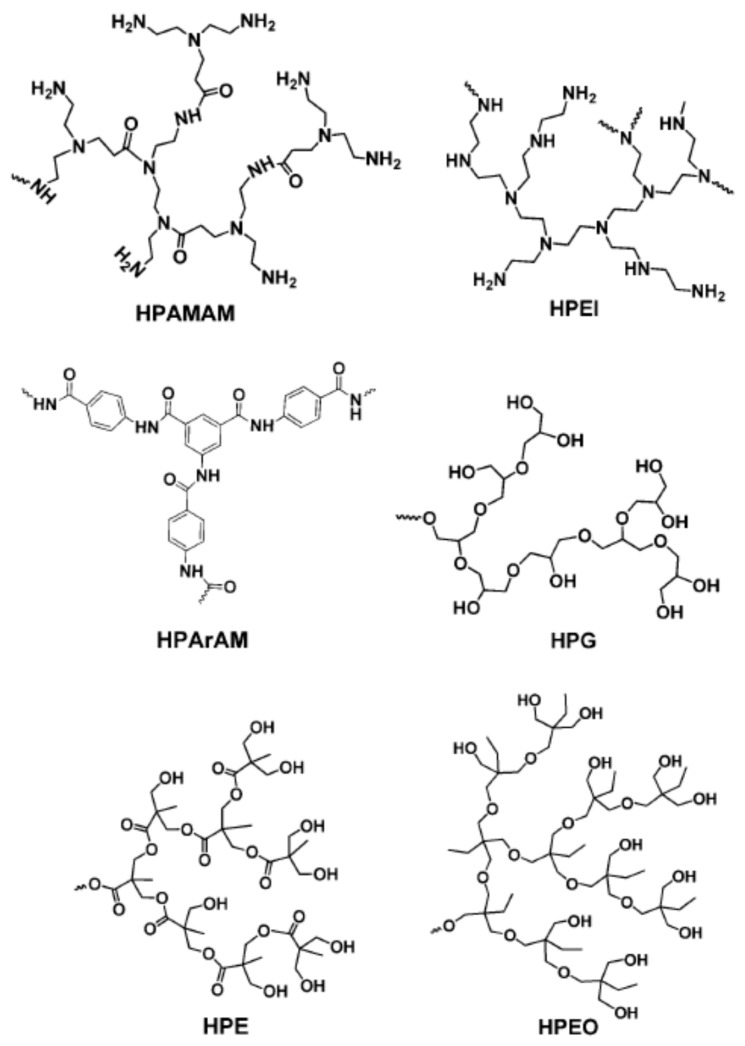
Schematic structures of classic HMs (and their acronyms) used as stabilizers to prepare NCs.

To date, six major kinds of HMs have been employed to prepare NCs. As shown in Figure 6, the acronyms of these HMs are hyperbranched polyamidoamines (HPAMAM) [86,87], hyperbranched poly(ethylene imine) (HPEI) [88,89,90], hyperbranched polyglycerol (HPG) [91,92,93], hyperbranched polyester (HPE) [94,95], hyperbranched poly(acryl amide) (HPAM) [96,97,98] and hyperbranched poly(ether polyols) (HPEO) [99]. Using these HMs as stabilizers, various semiconducting and metallic-conducting NCs have been prepared for diverse applications.

Most quantum dots (QDs) are synthesized using the ‘HMs first’ approach [88,89,90,100,101,102,103,104]. Hydroxyl-ended HPG (M_n_ > 20000 g mol^−1^) was directly used as the stabilizer to prepare QDs that included ZnS, Ag_2_S, PbS, CuS, and CdS [92]. Due to the role of HPG, various QDs displayed good solubility in water and DMF, and also showed low toxicity with good biocompatibility. Excluding unmodified HPGs, thioether-functionalized HPGs could be employed to prepare CdS and CdSe QDs [93]. Interestingly, the sizes of the resultant QDs depended on the molecular weights of the modified HPGs. In addition, the ligand-exchange strategy showed its superiority with regard to the size control of the NCs, because NC particles can be pre-formed. HPEI exchanged with hydrophobic surfactants of CdSe@ZnS QDs, can form very stable colloids in chloroform [105]. Compared with the aforementioned approaches, surface chemical grafting onto QDs is a more reliable way to stabilize NCs. Coating QDs with a protective shell can effectively avoid fluorescence quenching or the release of toxic metal ions [106,107,108,109].

Incidentally, multifarious factors, such as DB, the reaction temperature, and the concentration of metal ions, contribute to the particle size of NCs [110,111,112]. Other than monometallic (Au, Ag, Pt, Pd, and Ru) NCs, bimetallic (Au/Pt, Au/Pd, and Au/Ru) NCs [98] and smart HM-stabilized NCs [113] (thermo- or pH-responsive ones) have also easily been achieved using a similar strategy.

### 5.3. Bio-Applications

Similar to the amphiphilic linear block copolymers, amphiphilic HMs can be self-assembled into various supramolecular structures in solution or through interfacial self-assembly. Supramolecular structures have potential applications in biomedical areas, because of their biocompatibility and adjustable molecular architectures. Hyperbranched polyethers, polyesters, polyphosphates, and polysaccharides could be candidates for biomedical uses in areas including cytomimetic chemistry, drug delivery, gene transfection, antimicrobial material, and bio-imaging fields [114,115,116].

Compared with small molecular liposomes, the HM vesicles (HMVs) formed, display lower membrane fluidity and higher stability. HMVs can induce multivalent interactions among vesicles, like a biomembrane does. Moreover, the size of HMVs is very close to that of a cell, allowing direct observation through optical or fluorescent microscopy. Zhou and Yan revealed that membrane fusions were initiated even by small perturbations or by changing the osmotic pressure [117,118].

Apart from cytomimetic chemistry, supramolecular aggregates formed by HM self-assembly have been utilized to load drugs. Compared with naked drugs, HM-drug complexes can improve solubility and prolong service time. At the same time, they can easily penetrate cell membranes and selectively accumulate, as well as be retained, at tumor sites [119].

Cationic HMs (e.g., hyperbranched polyethylenimine, HPEI) mixed with electronegative DNA can form HM-DNA polyplexes for gene transfection. Compared with viral vectors, HMs displayed various advantages such as higher safety, weaker immune responses, more facile synthesis, and easier operation [120,121,122,123,124,125,126].

HMs have also been widely used as antibacterial/antifouling materials. Due to their good biocompatibility and chemical stability, HPGs are promising antifouling materials that can be employed to prevent the attachment of proteins [127].

In the bio-imaging field, HM-probe-conjugates with good water solubility and available functional groups are good solutions to problems associated with low quantum yield and poor specificity. Zhu and Yan grafted fluorescein isothiocyanate on peripheral hyperbranched polysulfonamine (HPSA) through the reaction of isothiocyanate and a primary amino group [128,129]. With low cytotoxicity and good serum compatibility, the HPSA-probe conjugate can be used for bio-imaging or for tracking cells [125].

**Figure 7:**
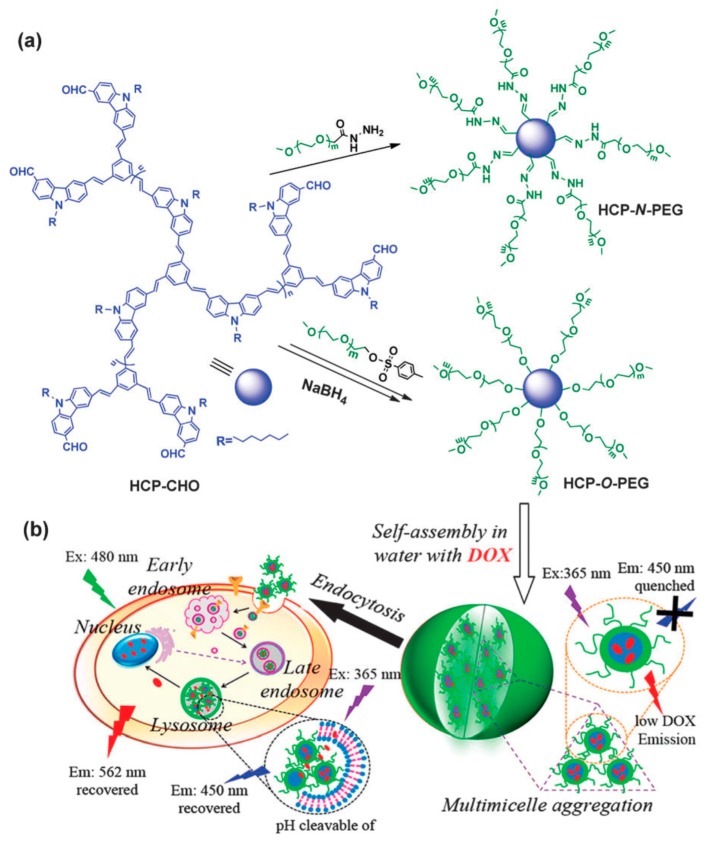
(**a**) Synthesis of HCP-*N*-PEG and HCP-*O*-PEG conjugated copolymers; (**b**) Self-assembly of conjugated copolymers and their endocytosis in tumor cells. Reproduced from [130] with permission from the American Chemical Society.

Star-like HMs (HCP-N-PEG and HCP-O-PEG) have an hyperbranched conjugated polymer (HCP) core and linear polyethylene glycol (PEG) arms. They showed superior fluorescein response sensitivity compared to that of small fluorophores, and could be used as drug carriers for tumor therapy (Figure 7) [130].

### 5.4. Carbon Nanomaterial/HM Nanocomposites

Because of their highly branched architecture, HMs have less intermolecular entanglement, which leads to good solubility, low viscosity, and unusual rheological properties. Their unique 3-D architecture offers enough steric hindrance to avoid aggregation of the nanoparticles. Therefore, HPs are good dispersants and surface modifiers for carbon nanomaterials, such as carbon nanotubes (CNTs) and graphene (or graphene nanoplatelets). 

**Figure 8:**
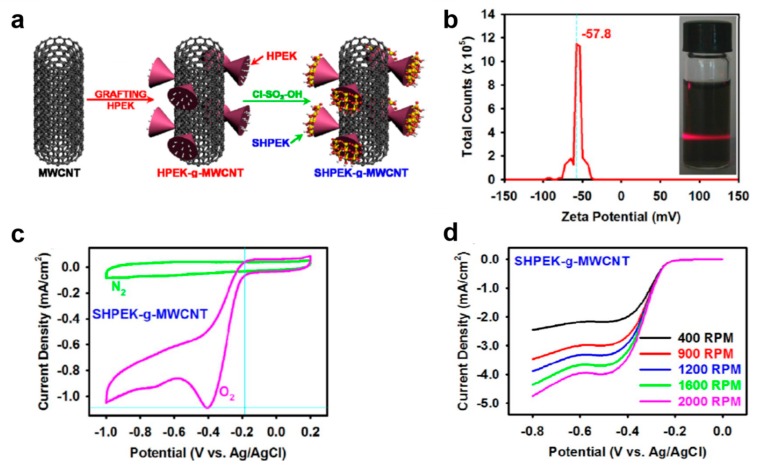
(**a**) Schematic demonstrations for SHPEK-g-MWCNT. (**b**) Zeta-potential curve of SHPEK-g-MWCNT (Inset: a photograph of the solution with hand-held laser shining). (**c**) Cyclic voltammograms in nitrogen- and oxygen-saturated 0.1 M aqueous KOH solution for SHPEK-g-MWCNT. (**d**) RDE voltammograms in oxygen-saturated 0.1 M aq. KOH solution with a scan rate of 0.01 V/s at different rotation rates. Reproduced from [131] with permission from the American Chemical Society.

When dendritic sulfonated hyperbranched poly-(ether-ketone) (SHPEK) was grafted onto the surfaces of multiwall carbon nanotubes (MWCNT or MWNT), the resultant nanocomposites (e.g., SHPEK-g-MWCNT) were easily dispersible in water (zeta potential of −57.8 mV; see Figure 8). SHPEK-g-MWCNT film showed sheet resistance as low as 63 Ω/sq and high electrocatalytic activity for the oxygen reduction reaction (ORR), without heteroatom doping onto the MWCNT framework [131]. 

Carbon nanomaterial/HM nanocomposites exhibited enhanced performance due to their favorable synergetic effects [132,133]. HMs exhibit low intrinsic viscosity, thus endowing the nanocomposites with good processability. There are two major methods for preparing nanocomposites or hybrids: (i) direct mixing of HMs with carbon nanomaterials and (ii) in situ polymerization of HMs in the presence of carbon nanomaterials. If HMs and carbon nanomaterials are linked by covalent bonds, the phase separation issue at the interface can be efficiently eliminated and the overall performance is greatly enhanced.

In the case of HPPS-g-MWCNT prepared from grafting of hyperbranched poly(phenyl sulfide) (HPPS) onto the surface of MWCNT, the dispersibility and melt-processability of the nanocomposite were significantly enhanced. Thus, the nanocomposite specimens could be easily compression-molded. Without chemical doping, the surface conductivities of as-prepared HPPS-g-MWNT film were in the semi-metallic transport region (3.56 S cm^−1^) [134].

**Figure 9:**
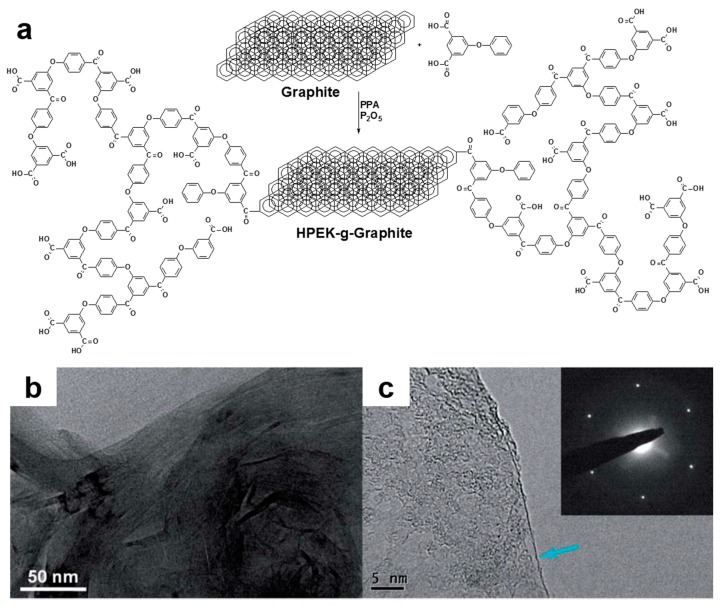
(**a**) ‘Direct’ Friedel-Crafts acylation reaction between graphite and HPEK in PPA/P_2_O_5_ medium. TEM images: (**b**) HPEK-g-graphite; (**c**) ‘Edge-on’ view (Inset: a selected area electron diffraction (SAED) pattern obtained from the basal area). Reproduced from [137] with permission from the Royal Society of Chemistry.

Graphene has attracted increasing attention and been subjected to rapid development because of its unique atom-thick 2-D structure and excellent properties. It has a wide range of promising potential applications [135,136]. Exfoliation of graphite to produce graphene could be achieved very simply by a wedge effect using HMs. In situ ‘direct’ grafting of HMs to the edges of pristine graphite could exfoliate graphitic layers to form graphene (Figure 9). Due to the 3-D molecular architectures of HMs, the solubility of HM grafted graphene is profoundly improved compared with grafting of its linear analogue. This result is because HM provides numerous polar peripheral groups that not only act as macromolecular wedges, but also exhibit chemical affinity for solvents [137]. 

**Figure 10:**
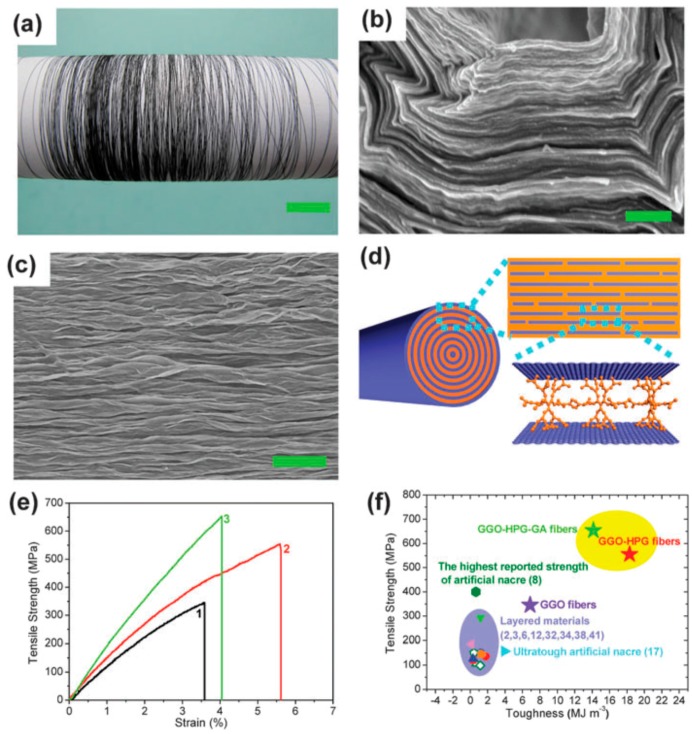
(**a**) Image of a 30 m long GO-HPG gel fiber (scale bar = 10 mm). (**b**,**c**) SEM images of a cross-section of a GO-HPG gel fiber ((**b**,**c**), scale bars of 250 nm and 3.0 mm, respectively); (**d**) Wet-spinning assembly of complex LCs into nacre-mimetic fibers with hierarchical structures; (**e**) Typical stress-strain curves: (1) GO only; (2) GO-HPG; (3) GO-HPG-GA; (**f**) The strain rate is 10% per minute. Reproduced from [140] with permission from Nature.

Graphene oxide (GO) possesses many available functional groups (e.g., hydroxyl and epoxide groups) on its basal area and along edges [138], which allow further chemical modification. Furthermore, these functional groups endow GO sheets with strong hydrophilicity, which makes GO fully dispersible in water or polar solvents (such as DMF and NMP) [139]. Through a liquid crystal self-templating methodology, next-generation continuous nacre-mimics with extreme strength and toughness have been achieved [140,141]. Hierarchically assembled fibers exhibited the highest tensile strength (652 MPa) and excellent ductility, with a toughness of 18 MJ m^−3^. The outstanding mechanical performance of GO-HPG fibers is ascribed to their hierarchically assembled structure and uniform alignment of GO sheets (Figure 10).

## 6. Conclusions and Outlook 

The major developments of synthetic strategies, the relationship between structures and properties, and many of the applications for HMs have been summarized in this paper. It is noteworthy that the development of applications for HMs is still in its infancy and further research is required to maximize their full potential. Moreover, because this is still an area of emerging research, some problems need to be solved, many knowledge gaps should be filled, and key limitations should be overcome. These include such as DB control, introduction of hetero-atoms, synthesis of HMs with 2D structure, development of sequence-controlled HMs, and biocompatibility.

## Figures and Tables

**Table 1:** Comparison of hyperbranched macromolecules with linear polymers and dendrimers. Reproduced from [7] with permission from the Royal Society of Chemistry.

	Linear	Hyperbranched	Dendrimer
Structure	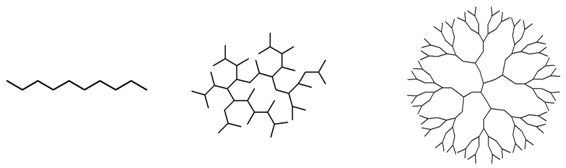		
Topology	1D, linear	3D, irregular	3D, regular
Synthesis	One-step, facile	One-step, relatively facile	Multi-step, laborious
Purification	Precipitation	Precipitation or classification	Chromatography
Scaling-up	Already, easy	Already, easy	Difficult
MW ^1^	Discrepant	Discrepant	Identical
PDI ^2^	>1.1	>1.1	1.0 (<1.05)
DB ^3^	0	0.4–0.6	1.0
Entanglement	Strong	Weak	Very weak or none
Viscosity	High	Low	Very low
Solubility	Low	High	High
Functional group	At two ends	At linear and terminal units	On periphery (terminal units)
Reactivity	Low	High	High
Strength	High	Low	Very low

^1^ Molecular weight; ^2^ Polydispersity index; ^3^ Degree of branching.

## References

[1] Jiang W., Zhou Y., Yan D. (2015). Hyperbranched polymer vesicles: From self-assembly, characterization, mechanisms, and properties to applications. Chem. Soc. Rev..

[2] Carminade A.-M., Yan D., Smith D.K. (2015). Dendrimers and hyperbranched polymers. Chem. Soc. Rev..

[3] Sun H.-J., Zhang S., Percec V.F. (2015). From structure to function via complex supramolecular dendrimer systems. Chem. Soc. Rev..

[4] Wu W., Tang R., Li Q., Li Z. (2015). Functional hyperbranched polymers with advanced optical, electrical and magnetic properties. Chem. Soc. Rev..

[5] Voit B.I., Lederer A. (2009). Hyperbranched and Highly Branched Polymer Architectures—Synthetic Strategies and Major Characterization Aspects. Chem. Rev..

[6] Carlmark A., Hawker C., Hult A., Malkoch M. (2009). New methodologies in the construction of dendritic materials. Chem. Soc. Rev..

[7] Zheng Y., Li S., Weng Z., Gao C. (2015). Hyperbranched polymers: Advances from synthesis to applications. Chem. Soc. Rev..

[8] Jikei M., Kakimoto M.-A. (2001). Hyperbranched polymers: A promising new class of materials. Prog. Polym. Sci..

[9] Cook A.B., Barbey R., Burns J.A., Perrier S. (2016). Hyperbranched Polymers with High Degrees of Branching and Low Dispersity Values: Pushing the Limits of Thiol–Yne Chemistry. Macromolecules.

[10] Sun J., Aly K.I., Kuckling D. (2017). A novel one-pot process for the preparation of linear and hyperbranched polycarbonates of various diols and triols using dimethyl carbonate. RSC Adv..

[11] Kim Y.H., Webster O.W. (1992). Hyperbranched polyphenylenes. Macromolecules.

[12] Aydogan C., Yilmaz G., Yagci Y., Morgenroth F. (2017). Synthesis of Hyperbranched Polymers by Photoinduced Metal-Free ATRP. Macromolecules.

[13] Khalyavina A., Schallausky F., Komber H., Samman M.A., Radke W., Lederer A. (2010). Aromatic–Aliphatic Polyesters with Tailored Degree of Branching Based on AB/AB_2_ and ABB*/AB_2_ Monomers. Macromolecules.

[14] Xue Z., Finke A.D., Moore J.S. (2010). Synthesis of Hyperbranched Poly(m-phenylene)s via Suzuki Polycondensation of a Branched AB_2_ Monomer. Macromolecules.

[15] Yamaguchi N., Wang J.-S., Hewitt J.M., Lenhart W.C., Mourey T.H. (2002). Acid chloride-functionalized hyperbranched polyester for facile and quantitative chain-end modification: One-pot synthesis and structure characterization. J. Polym. Sci. Part A Polym. Chem..

[16] Mahdavi H., Shahalizade T. (2015). Preparation, characterization and performance study of cellulose acetate membranes modified by aliphatic hyperbranched polyester. J. Membr. Sci..

[17] Niu S., Yan H., Chen Z., Li S., Xu P., Zhi X. (2016). Unanticipated bright blue fluorescence produced from novel hyperbranched polysiloxanes carrying unconjugated carbon–carbon double bonds and hydroxyl groups. Polym. Chem..

[18] Ye L., Letchford K., Heller M., Liggins R., Guan D., Kizhakkedathu J.N., Brooks D.E., Jackson J.K., Burt H.M. (2011). Synthesis and Characterization of Carboxylic Acid Conjugated, Hydrophobically Derivatized, Hyperbranched Polyglycerols as Nanoparticulate Drug Carriers for Cisplatin. Biomacromolecules.

[19] Perala S.K., Ramakrishnan S. (2017). Effect of Spacer Stiffness on the Properties of Hyperbranched Polymers. Macromolecules.

[20] Fréchet J.M.J., Henmi M., Gitsov I., Aoshima S., Leduc M.R., Grubbs R.B. (1995). Self-Condensing Vinyl Polymerization: An Approach to Dendritic Materials. Science.

[21] Higashihara T., Segawa Y., Sinananwanich W., Ueda M. (2011). Synthesis of hyperbranched polymers with controlled degree of branching. Polym. J..

[22] Satoh Y., Miyachi K., Matsuno H., Isono T., Tajima K., kakuchi T., Satoh T. (2016). Synthesis of Well-Defined Amphiphilic Star-Block and Miktoarm Star Copolyethers via t-Bu-P4-Catalyzed Ring-Opening Polymerization of Glycidyl Ethers. Macromolecules.

[23] Rannard S., Davis N., McFarland H. (2000). Synthesis of dendritic polyamides using novel selective chemistry. Polym. Int..

[24] Choi J.-Y., Oh S.-J., Lee H.-J., Wang D.H., Tan L.-S., Baek J.-B. (2007). In-Situ Grafting of Hyperbranched Poly(ether ketone)s onto Multiwalled Carbon Nanotubes via the A_3_ + B_2_ Approach. Macromolecules.

[25] Masukawa S., Kikkawa T., Fujimori A., Oishi Y., Shibasaki Y. (2015). Synthesis of a A2B3-type Hyperbranched Copolymers Based on a 3-Armed Unimolecular 4-N-Methylbenzamide Pentamer and Poly(propylene oxide). Chem. Lett..

[26] Tomalia D.A., Hedstrand D.M., Ferritto M.S. (1991). Comb-burst dendrimer topology: New macromolecular architecture derived from dendritic grafting. Macromolecules.

[27] Nguyen C., Hawker C.J., Miller R.D., Huang E., Hedrick J.L., Gauderon R., Hilborn J.G. (2000). Hyperbranched Polyesters as Nanoporosity Templating Agents for Organosilicates. Macromolecules.

[28] Zhou Y., Huang W., Liu J., Zhu X., Yan D. (2010). Self-Assembly of Hyperbranched Polymers and Its Biomedical Applications. Adv. Mater..

[29] Mourey T.H., Turner S.R., Rubinstein M., Frechet J.M.J., Hawker C.J., Wooley K.L. (1992). Unique behavior of dendritic macromolecules: Intrinsic viscosity of polyether dendrimers. Macromolecules.

[30] Ohta Y., Sakurai K., Matsuda J., Yokozawa T. (2016). Chain-growth condensation polymerization of 5-aminoisophthalic acid triethylene glycol ester to afford well-defined, water-soluble, thermoresponsive hyperbranched polyamides. Polymer.

[31] Chen H., Kong J. (2016). Hyperbranched polymers from A2 + B3 strategy: Recent advances in description and control of fine topology. Polym. Chem..

[32] Zigmond J.S., Pavia-Sanders A., Russell J.D., Wooley K.L., Percec V. (2016). Dynamic Anti-Icing Coatings: Complex, Amphiphilic Hyperbranched Fluoropolymer Poly(ethylene glycol) Cross-Linked Networks with an Integrated Liquid Crystalline Comonomer. Chem. Mater..

[33] Zigmond J.S., Letteri R.A., Wooley K.L., Percec V. (2016). Amphiphilic Cross-Linked Liquid Crystalline Fluoropolymer-Poly(ethylene glycol) Coatings for Application in Challenging Conditions: Comparative Study between Different Liquid Crystalline Comonomers and Polymer Architectures. ACS Appl. Energy Mater..

[34] Wang G.-W., Chen B., Zhuang L.-H., Yun K., Guo J.-R., Wang Y., Xu B. (2015). Dyeing performances of ramie fabrics modified with an amino-terminated aliphatic hyperbranched polymer. Cellulose.

[35] Kim Y.H., Beckerbauer R. (1994). Role of End Groups on the Glass Transition of Hyperbranched Polyphenylene and Triphenylbenzene Derivatives. Macromolecules.

[36] Wu C., Huang X., Wang G., Wu X., Yang K., Li S., Jiang P. (2012). Hyperbranched-polymer functionalization of graphene sheets for enhanced mechanical and dielectric properties of polyurethane composites. J. Mater. Chem..

[37] Lei X., Chen Y., Qiao M., Tian L., Zhang Q., Romagnoli B. (2016). Hyperbranched polysiloxane (HBPSi)-based polyimide films with ultralow dielectric permittivity, desirable mechanical and thermal properties. J. Mater. Chem. C.

[38] Gadwal I., Binder S., Stuparu M.C., Khan A. (2014). Dual-Reactive Hyperbranched Polymer Synthesis through Proton Transfer Polymerization of Thiol and Epoxide Groups. Macromolecules.

[39] Hölter D., Burgath A., Frey H. (1997). Degree of branching in hyperbranched polymers. Acta Polym..

[40] Kambouris P., Hawker C.J. (1993). A versatile new method for structure determination in hyperbranched macromolecules. J. Chem. Soc. Perkin Trans. 1.

[41] Zhu X., Zhou Y., Yan D. (2011). Influence of branching architecture on polymer properties. J. Polym. Sci. Part B Polym. Phys..

[42] Spears B.R., Waksal J., mcQuade C., Lanier L., Harth E. (2013). Controlled branching of polyglycidol and formation of protein–glycidol bioconjugates via a graft-from approach with “PEG-like” arms. Chem. Commun..

[43] Unal S., Lin Q., Mourey T.H., Long T.E. (2005). Tailoring the Degree of Branching: Preparation of Poly(ether ester)s via Copolymerization of Poly(ethylene glycol) Oligomers (A2) and 1,3,5-Benzenetricarbonyl Trichloride (B3). Macromolecules.

[44] Unal S., Oguz C., Yilgor E., Gallivan M., Long T.E., Yilgor I. (2005). Understanding the structure development in hyperbranched polymers prepared by oligomeric A2 + B3 approach: Comparison of experimental results and simulations. Polymer.

[45] Schubert C., Schömer M., Steube M., Decker S., Friedrich C., Frey H. (2018). Systematic Variation of the Degree of Branching (DB) of Polyglycerol via Oxyanionic Copolymerization of Glycidol with a Protected Glycidyl Ether and Its Impact on Rheological Properties. Macromol. Chem. Phys..

[46] Segawa Y., Higashihara T., Ueda M. (2013). Synthesis of hyperbranched polymers with controlled structure. Polym. Chem..

[47] Mai Y., Zhou Y., Yan D., Lu H. (2003). Effect of Reaction Temperature on Degree of Branching in Cationic Polymerization of 3-Ethyl-3-(hydroxymethyl)oxetane. Macromolecules.

[48] Popeney C.S., Lukowiak M.C., Böttcher C., Schade B., Welker P., Mangoldt D., Gunkel G., Guan Z., Haag R. (2012). Tandem Coordination, Ring-Opening, Hyperbranched Polymerization for the Synthesis of Water-Soluble Core–Shell Unimolecular Transporters. ACS Macro Lett..

[49] Shi Y., Graff R.W., Cao X., Wang X., Gao H. (2015). Chain-Growth Click Polymerization of AB_2_ Monomers for the Formation of Hyperbranched Polymers with Low Polydispersities in a One-Pot Process. Angew. Chem. Int. Ed..

[50] Segawa Y., Higashihara T., Ueda M. (2010). Hyperbranched Polymers with Controlled Degree of Branching from 0 to 100%. J. Am. Chem. Soc..

[51] Guan Z. (2010). Recent Progress of Catalytic Polymerization for Controlling Polymer Topology. Chem. Asian J..

[52] Guan Z. (1999). Chain Walking: A New Strategy to Control Polymer Topology. Science.

[53] Chen L., Zhu X., Yan D., Chen Y., Chen Q., Yao Y. (2006). Controlling Polymer Architecture through Host-Guest Interactions. Angew. Chem. Int. Ed..

[54] Liu N., Vignolle J., Vincent J.-M., Robert F., Landais Y., Cramail H., Taton D. (2014). One-Pot Synthesis and PEGylation of Hyperbranched Polyacetals with a Degree of Branching of 100%. Macromolecules.

[55] Radke W., Litvinenko G., Müller A.H.E. (1998). Effect of Core-Forming Molecules on Molecular Weight Distribution and Degree of Branching in the Synthesis of Hyperbranched Polymers. Macromolecules.

[56] Ishida Y., Sun A.C.F., Jikei M., Kakimoto M.-A. (2000). Synthesis of Hyperbranched Aromatic Polyamides Starting from Dendrons as ABx Monomers: Effect of Monomer Multiplicity on the Degree of Branching. Macromolecules.

[57] Lach C., Frey H. (1998). Enhancing the Degree of Branching of Hyperbranched Polymers by Postsynthetic Modification. Macromolecules.

[58] Huang W., Su L., Bo Z. (2009). Hyperbranched Polymers with a Degree of Branching of 100% Prepared by Catalyst Transfer Suzuki–Miyaura Polycondensation. J. Am. Chem. Soc..

[59] Zhou Z., Yan D. (1999). Kinetic analysis for polycondensation of AB (g) type monomers. Chem. J. Chin. Univ. Chin..

[60] Cao X., Shi Y., Wang X., Graff R.W., Gao H. (2016). Design a Highly Reactive Trifunctional Core Molecule To Obtain Hyperbranched Polymers with over a Million Molecular Weight in One-Pot Click Polymerization. Macromolecules.

[61] Cheng K.-C., Chuang T.-H., Chang J.-S., Guo W., Su W.-F. (2005). Effect of Feed Rate on Structure of Hyperbranched Polymers Formed by Self-Condensing Vinyl Polymerization in Semibatch Reactor. Macromolecules.

[62] Cheng K.-C. (2003). Effect of feed rate on structure of hyperbranched polymers formed by stepwise addition of AB_2_ monomers into multifunctional cores. Polymer.

[63] Möck A., Burgath A., Hanselmann R., Frey H. (2001). Synthesis of Hyperbranched Aromatic Homo- and Copolyesters via the Slow Monomer Addition Method. Macromolecules.

[64] Zhou Z., Jia Z., Yan D. (2010). Effect of slow monomer addition on molecular parameters of hyperbranched polymers synthesized in the presence of multifunctional core molecules. Sci. China Chem..

[65] Cheng K.-C., Lai W.-J. (2017). Effect of feed rate of end-capping molecules on structure of hyperbranched polymers formed from monomers A2 and B4 in semibatch process. Eur. Polym. J..

[66] Tobita H. (2014). Markovian Approach to Self-Condensing Vinyl Polymerization: Distributions of Molecular Weights, Degrees of Branching, and Molecular Dimensions. Macromol. Theory Simul..

[67] Litvinenko G.I., Müller A.H.E. (2002). Molecular Weight Averages and Degree of Branching in Self-Condensing Vinyl Copolymerization in the Presence of Multifunctional Initiators. Macromolecules.

[68] Gao C., Yan D., Frey H. (2011). Promising Dendritic Materials: An Introduction to Hyperbranched Polymers. Hyperbranched Polymers: Synthesis, Properties, and Applications.

[69] Li Z., Li Q., Qin J. (2011). Some new design strategies for second-order nonlinear optical polymers and dendrimers. Polym. Chem..

[70] Wu W., Huang L., Xiao L., Huang Q., Tang R., Ye C., Qin J., Li Z. (2012). New second-order nonlinear optical (NLO) hyperbranched polymers containing isolation chromophore moieties derived from one-pot “A2 + B4” approach via Suzuki coupling reaction. RSC Adv..

[71] Zhu Z., Li Z.A., Tan Y., Li Z., Li Q., Zeng Q., Ye C., Qin J. (2006). New hyperbranched polymers containing second-order nonlinear optical chromophores: Synthesis and nonlinear optical characterization. Polymer.

[72] Bai Y., Song N., Gao J.P., Sun X., Wang X., Yu G., Wang Z.Y. (2005). A New Approach to Highly Electrooptically Active Materials Using Cross-Linkable, Hyperbranched Chromophore-Containing Oligomers as a Macromolecular Dopant. J. Am. Chem. Soc..

[73] Li Z., Qin A., Lam J.W.Y., Dong Y., Dong Y., Ye C., Williams I.D., Tang B.Z. (2006). Facile Synthesis, Large Optical Nonlinearity, and Excellent Thermal Stability of Hyperbranched Poly(aryleneethynylene)s Containing Azobenzene Chromophores. Macromolecules.

[74] Scarpaci A., Blart E., Montembault V.R., Fontaine L., Rodriguez V., Odobel F. (2009). Synthesis and Nonlinear Optical Properties of a Peripherally Functionalized Hyperbranched Polymer by DR1 Chromophores. ACS Appl. Mater. Interfaces.

[75] Scarpaci A., Blart E., Montembault V., Fontaine L., Rodriguez V., Odobel F. (2009). A new crosslinkable system based on thermal Huisgen reaction to enhance the stability of electro-optic polymers. Chem. Commun..

[76] Li J., Bo Z. (2004). “AB_2_ + AB” Approach to Hyperbranched Polymers Used as Polymer Blue Light Emitting Materials. Macromolecules.

[77] Xin Y., Wen G.-A., Zeng W.-J., Zhao L., Zhu X.-R., Fan Q.-L., Feng J.-C., Wang L.-H., Peng B., Cao Y. (2005). Hyperbranched Oxadiazole-Containing Polyfluorenes: Toward Stable Blue Light PLEDs. Macromolecules.

[78] Tsai L.-R., Chen Y. (2007). Novel Hyperbranched Polyfluorenes Containing Electron-Transporting Aromatic Triazole as Branch Unit. Macromolecules.

[79] Cao X.-Y., Zhou X.-H., Zi H., Pei J. (2004). Novel Blue-Light-Emitting Truxene-Containing Hyperbranched and Zigzag Type Copolymers: Synthesis, Optical Properties, and Investigation of Thermal Spectral Stability. Macromolecules.

[80] Wu Y., Hao X., Wu J., Jin J., Ba X. (2010). Pure Blue-Light-Emitting Materials: Hyperbranched Ladder-Type Poly(p-phenylene)s Containing Truxene Units. Macromolecules.

[81] Wu G., Yang Y., He C., Chen X., Li Y. (2008). A new triphenylamine-based hyperbranched polyfluorene with oxadiazole units on its side chains. Eur. Polym. J..

[82] Li Z.A., Ye S., Liu Y., Yu G., Wu W., Qin J., Li Z. (2010). New Hyperbranched Conjugated Polymers Containing Hexaphenylbenzene and Oxadiazole Units: Convenient Synthesis and Efficient Deep Blue Emitters for PLEDs Application. J. Phys. Chem. B.

[83] Liu J., Yu L., Zhong C., He R., Yang W., Wu H., Cao Y. (2012). Highly efficient green-emitting electrophosphorescent hyperbranched polymers using a bipolar carbazole-3,6-diyl-co-2,8-octyldibenzothiophene-S,S-dioxide-3,7-diyl unit as the branch. RSC Adv..

[84] Hu X., Zhou L., Gao C. (2011). Hyperbranched polymers meet colloid nanocrystals: A promising avenue to multifunctional, robust nanohybrids. Colloid Polym. Sci..

[85] Zhu Q., Qiu F., Zhu B., Zhu X. (2013). Hyperbranched polymers for bioimaging. RSC Adv..

[86] Pérignon N., Marty J.-D., Mingotaud A.-F., Dumont M., Rico-Lattes I., Mingotaud C. (2007). Hyperbranched Polymers Analogous to PAMAM Dendrimers for the Formation and Stabilization of Gold Nanoparticles. Macromolecules.

[87] Saliba S., Valverde Serrano C., Keilitz J., Kahn M.L., Mingotaud C., Haag R., Marty J.-D. (2010). Hyperbranched Polymers for the Formation and Stabilization of ZnO Nanoparticles. Chem. Mater..

[88] Tuchbreiter L., Mecking S. (2007). Hydroformylation with Dendritic-Polymer-Stabilized Rhodium Colloids as Catalyst Precursors. Macromol. Chem. Phys..

[89] Gladitz M., Reinemann S., Radusch H.-J. (2009). Preparation of Silver Nanoparticle Dispersions via a Dendritic-Polymer Template Approach and their Use for Antibacterial Surface Treatment. Macromol. Mater. Eng..

[90] Krämer M., Pérignon N., Haag R., Marty J.-D., Thomann R., Lauth-de Viguerie N., Mingotaud C. (2005). Water-Soluble Dendritic Architectures with Carbohydrate Shells for the Templation and Stabilization of Catalytically Active Metal Nanoparticles. Macromolecules.

[91] Shen Z., Duan H., Frey H. (2007). Water-Soluble Fluorescent Ag Nanoclusters Obtained from Multiarm Star Poly(acrylic acid) as “Molecular Hydrogel” Templates. Adv. Mater..

[92] Zhou L., Gao C., Hu X., Xu W. (2011). General Avenue to Multifunctional Aqueous Nanocrystals Stabilized by Hyperbranched Polyglycerol. Chem. Mater..

[93] Wan D., Fu Q., Huang J. (2006). Synthesis of a thioether modified hyperbranched polyglycerol and its template effect on fabrication of CdS and CdSe nanoparticles. J. Appl. Polym. Sci..

[94] Wei X., Zhu B., Xu Y. (2005). Preparation and stability of copper particles formed using the template of hyperbranched poly(amine-ester). Colloid Polym. Sci..

[95] Zhao Y., Zou J., Shi W. (2005). Synthesis and characterization of PbS/modified hyperbranched polyester nanocomposite hollow spheres at room temperature. Mater. Lett..

[96] Monticelli O., Russo S., Campagna R., Voit B. (2005). Preparation and characterisation of blends based on polyamide 6 and hyperbranched aramids as palladium nanoparticle supports. Polymer.

[97] Kakati N., Mahapatra S.S., Karak N. (2008). Silver Nanoparticles in Polyacrylamide and Hyperbranched Polyamine Matrix. J. Macromol. Sci. Part A Pure Appl. Chem..

[98] Mahapatra S.S., Karak N. (2008). Silver nanoparticle in hyperbranched polyamine: Synthesis, characterization and antibacterial activity. Mater. Chem. Phys..

[99] Richter T.V., Schüler F., Thomann R., Mülhaupt R., Ludwigs S. (2009). Nanocomposites of Size-Tunable ZnO-Nanoparticles and Amphiphilic Hyperbranched Polymers. Macromol. Rapid Commun..

[100] Sun Y., Liu Y., Guizhe Z., Zhang Q. (2007). Effects of hyperbranched poly(amido-amine)s structures on synthesis of Ag particles. J. Appl. Polym. Sci..

[101] Drozd M., Pietrzak M., Parzuchowski P., Mazurkiewicz-Pawlicka M., Malinowka E. (2015). Peroxidase-like activity of gold nanoparticles stabilized by hyperbranched polyglycidol derivatives over a wide pH range. Nanotechnology.

[102] Zhu L., Shi Y., Tu C., Wang R., Pang Y., Qiu F., Zhu X., Yan D., He L., Jin C. (2010). Construction and Application of a pH-Sensitive Nanoreactor via a Double-Hydrophilic Multiarm Hyperbranched Polymer. Langmuir.

[103] Keilitz J., Radowski M.R., Marty J.-D., Haag R., Gauffre F., Mingotaud C. (2008). Dendritic Polymers with a Core–Multishell Architecture: A Versatile Tool for the Stabilization of Nanoparticles. Chem. Mater..

[104] Moisan S., Martinez V., Weisbecker P., Cansell F., Mecking S., Aymonier C. (2007). General Approach for the Synthesis of Organic–Inorganic Hybrid Nanoparticles Mediated by Supercritical CO_2_. J. Am. Chem. Soc..

[105] Chen Y., Frey H., Thomann R., Stiriba S.-E. (2006). Optically active amphiphilic hyperbranched polyglycerols as templates for palladium nanoparticles. Inorg. Chim. Acta.

[106] Zhou L., Gao C., Hu X., Xu W. (2010). One-Pot Large-Scale Synthesis of Robust Ultrafine Silica-Hybridized CdTe Quantum Dots. ACS Appl. Mater. Interfaces.

[107] Zhou L., Gao C., Xu W. (2010). Magnetic Dendritic Materials for Highly Efficient Adsorption of Dyes and Drugs. ACS Appl. Mater. Interfaces.

[108] Zhou L., Gao C., Xu W., Wang X., Xu Y. (2009). Enhanced Biocompatibility and Biostability of CdTe Quantum Dots by Facile Surface-Initiated Dendritic Polymerization. Biomacromolecules.

[109] Shi Y., Du J., Zhou L., Li X., Zhou Y., Li L., Zang X., Zhang X., Pan F., Zhang H. (2012). Size-controlled preparation of magnetic iron oxidenanocrystals within hyperbranched polymers and their magnetofection in vitro. J. Mater. Chem..

[110] Yu B., Jiang X., Yin J. (2013). Responsive hybrid nanosheets of hyperbranched poly(ether amine) as a 2D-platform for metal nanoparticles. Chem. Commun..

[111] Zhou Y., Yan D. (2009). Supramolecular self-assembly of amphiphilic hyperbranched polymers at all scales and dimensions: Progress, characteristics and perspectives. Chem. Commun..

[112] Dey P., Blakey I., Thurecht K.J., Fredericks P.M. (2013). Self-Assembled Hyperbranched Polymer–Gold Nanoparticle Hybrids: Understanding the Effect of Polymer Coverage on Assembly Size and SERS Performance. Langmuir.

[113] Shen Y., Kuang M., Shen Z., Nieberle J., Duan H., Frey H. (2008). Gold Nanoparticles Coated with a Thermosensitive Hyperbranched Polyelectrolyte: Towards Smart Temperature and pH Nanosensors. Angew. Chem. Int. Ed..

[114] Jin H., Huang W., Zhu X., Zhou Y., Yan D. (2012). Biocompatible or biodegradable hyperbranched polymers: From self-assembly to cytomimetic applications. Chem. Soc. Rev..

[115] Wang D., Chen H., Su Y., Qiu F., Zhu L., Huan X., Zhu B., Yan D., Guo F., Zhu X. (2013). Supramolecular amphiphilic multiarm hyperbranched copolymer: Synthesis, self-assembly and drug delivery applications. Polym. Chem..

[116] Hartlieb M., Floyd T., Cook A.B., Sanchez-Cano C., Catrouillet S., Burns J.A., Perrier S. (2017). Well-defined hyperstar copolymers based on a thiol–yne hyperbranched core and a poly(2-oxazoline) shell for biomedical applications. Polym. Chem..

[117] Zhou Y., Yan D. (2005). Real-Time Membrane Fusion of Giant Polymer Vesicles. J. Am. Chem. Soc..

[118] Zhou Y., Yan D. (2005). Real-Time Membrane Fission of Giant Polymer Vesicles. Angew. Chem. Int. Ed..

[119] Gregory A., Stenzel M.H. (2012). Complex polymer architectures via RAFT polymerization: From fundamental process to extending the scope using click chemistry and nature’s building blocks. Prog. Polym. Sci..

[120] Wang X., He Y., Wu J., Gao C., Xu Y. (2010). Synthesis and Evaluation of Phenylalanine-Modified Hyperbranched Poly(amido amine)s as Promising Gene Carriers. Biomacromolecules.

[121] Ren Y., Jiang X., Pan D., Mao H.-Q. (2010). Charge Density and Molecular Weight of Polyphosphoramidate Gene Carrier Are Key Parameters Influencing Its DNA Compaction Ability and Transfection Efficiency. Biomacromolecules.

[122] Newland B., Tai H., Zheng Y., Velasco D., Di Luca A., Howdle S.M., Alexander C., Wang W., Pandit A. (2010). A highly effective gene delivery vector—Hyperbranched poly(2-(dimethylamino)ethyl methacrylate) from in situ deactivation enhanced ATRP. Chem. Commun..

[123] Chen M., Wu J., Zhou L., Jin C., Tu C., Zhu B., Wu F., Zhu Q., Zhu X., Yan D. (2011). Hyperbranched glycoconjugated polymer from natural small molecule kanamycin as a safe and efficient gene vector. Polym. Chem..

[124] Tu C., Li N., Zhu L., Zhou L., Su Y., Li P., Zhu X. (2013). Cationic long-chain hyperbranched poly(ethylene glycol)s with low charge density for gene delivery. Polym. Chem..

[125] Yu S., Chen J., Dong R., Su Y., Ji B., Zhou Y., Zhu X., Yan D. (2012). Enhanced gene transfection efficiency of PDMAEMA by incorporating hydrophobic hyperbranched polymer cores: Effect of degree of branching. Polym. Chem..

[126] Wang G., Yin H., Yin Ng J.C., Cai L., Li J., Tang B.Z., Liu B. (2013). Polyethyleneimine-grafted hyperbranched conjugated polyelectrolytes: Synthesis and imaging of gene delivery. Polym. Chem..

[127] Siegers C., Biesalski M., Haag R. (2004). Self-Assembled Monolayers of Dendritic Polyglycerol Derivatives on Gold That Resist the Adsorption of Proteins. Chem. Eur. J..

[128] Chen S., Tan Z., Li N., Wang R., He L., Shi Y., Jiang L., Li P., Zhu X. (2011). Highly Efficient Intracellular Drug Delivery with a Negatively Charged Hyperbranched Polysulfonamine. Macromol. Biosci..

[129] Qiu W., Xu J., Li X., Zhong L., Li J., Li J., Nan F. (2009). Design and Synthesis of Matrix Metalloprotease Photoaffinity Trimodular Probes. Chin. J. Chem..

[130] Qiu F., Wang D., Zhu Q., Zhu L., Tong G., Lu Y., Yan D., Zhu X. (2014). Real-Time Monitoring of Anticancer Drug Release with Highly Fluorescent Star-Conjugated Copolymer as a Drug Carrier. Biomacromolecules.

[131] Sohn G.-J., Choi H.-J., Jeon I.-Y., Chang D.W., Dai L., Baek J.-B. (2012). Water-Dispersible, Sulfonated Hyperbranched Poly(ether-ketone) Grafted Multiwalled Carbon Nanotubes as Oxygen Reduction Catalysts. ACS Nano.

[132] Caruso F. (2001). Nanoengineering of Particle Surfaces. Adv. Mater..

[133] Hood M., Mari M., Muñoz-Espí R. (2014). Synthetic Strategies in the Preparation of Polymer/Inorganic Hybrid Nanoparticles. Materials.

[134] Jeon I.-Y., Lee H.-J., Choi Y.S., Tan L.-S., Baek J.-B. (2008). Semimetallic Transport in Nanocomposites Derived from Grafting of Linear and Hyperbranched Poly(phenylene sulfide)s onto the Surface of Functionalized Multi-Walled Carbon Nanotubes. Macromolecules.

[135] Novoselov K.S. (2004). Electric Field Effect in Atomically Thin Carbon Films. Science.

[136] Roldán R., Chirolli L., Prada E., Silva-Guillén J.A., San-Jose P., Guinea F. (2017). Theory of 2D crystals: Graphene and beyond. Chem. Soc. Rev..

[137] Jeon I.-Y., Choi H.-J., Bae S.-Y., Chang D.W., Baek J.-B. (2011). Wedging graphite into graphene and graphene-like platelets by dendritic macromolecules. J. Mater. Chem..

[138] Zhu Y., Murali S., Cai W., Li X., Suk J.W., Potts J.R., Ruoff R.S. (2010). Graphene and Graphene Oxide: Synthesis, Properties, and Applications. Adv. Mater..

[139] Hirata M., Gotou T., Ohba M. (2005). Thin-film particles of graphite oxide. Preliminary studies for internal micro fabrication of single particle and carbonaceous electronic circuits. Carbon.

[140] Hu X., Xu Z., Liu Z., Gao C. (2013). Liquid crystal self-templating approach to ultrastrong and tough biomimic composites. Sci. Rep..

[141] Xu Z., Gao C. (2014). Graphene in Macroscopic Order: Liquid Crystals and Wet-Spun Fibers. Acc. Chem. Res..

